# The Evolution of Word Composition in Metazoan Promoter Sequence

**DOI:** 10.1371/journal.pcbi.0020150

**Published:** 2006-11-03

**Authors:** Eliot C Bush, Bruce T Lahn

**Affiliations:** 1 Department of Human Genetics, University of Chicago, Chicago, Illinois, United States of America; 2 Howard Hughes Medical Institute, University of Chicago, Chicago, Illinois, United States of America; University of Texas, United States of America

## Abstract

The field of molecular evolution provides many examples of the principle that molecular differences between species contain information about evolutionary history. One surprising case can be found in the frequency of short words in DNA: more closely related species have more similar word compositions. Interest in this has often focused on its utility in deducing phylogenetic relationships. However, it is also of interest because of the opportunity it provides for studying the evolution of genome function. Word-frequency differences between species change too slowly to be purely the result of random mutational drift. Rather, their slow pattern of change reflects the direct or indirect action of purifying selection and the presence of functional constraints. Many such constraints are likely to exist, and an important challenge is to distinguish them. Here we develop a method to do so by isolating the effects acting at different word sizes. We apply our method to 2-, 4-, and 8-base-pair (bp) words across several classes of noncoding sequence. Our major result is that similarities in 8-bp word frequencies scale with evolutionary time for regions immediately upstream of genes. This association is present although weaker in intronic sequence, but cannot be detected in intergenic sequence using our method. In contrast, 2-bp and 4-bp word frequencies scale with time in all classes of noncoding sequence. These results suggest that different genomic processes are involved at different word sizes. The pattern in 2-bp and 4-bp words may be due to evolutionary changes in processes such as DNA replication and repair, as has been suggested before. The pattern in 8-bp words may reflect evolutionary changes in gene-regulatory machinery, such as changes in the frequencies of transcription-factor binding sites, or in the affinity of transcription factors for particular sequences.

## Synopsis

One of the foundations of molecular evolution is the idea that more closely related species are more similar on the molecular level. One example that has been known for several years is the genomic composition of short words (i.e., short segments) of DNA. Given a sample of genome sequence, one can count the occurrences of all words of a certain length. It turns out that closely related species have more similar word frequencies. The pattern of how these frequencies change over evolutionary time is likely to be influenced by the many functions of the genome (coding for proteins, controlling gene expression, etc.). Bush and Lahn investigated the influence of genomic function on word-frequency variation in 13 animal genomes. Using a method designed to isolate the effects acting at particular word sizes, the authors examined how word frequencies vary in different categories of noncoding sequence. They found that interspecies patterns of word-frequency variation change depending on word size and sequence category. These results suggest that noncoding sequence is subject to different functional constraints depending on its location in the genome. An especially interesting possibility is that the patterns in longer words may reflect evolutionary changes in gene regulatory machinery.

## Introduction

Biological macromolecules accumulate changes over evolutionary time. Because of this, molecular differences between species contain information about evolutionary history [1]. Frequently this principle is used in the comparison of aligned protein or DNA sequences. However, it can also be observed at other levels of organization such as the ordering of genes on chromosomes [2] and the intron–exon structure of genes [3].

Another level of organization where this has been studied is the composition of short words in genomic DNA. Di- and trinucleotide frequencies are more similar in more closely related species [4,5]. Phylogenetic patterns like this have also been found using longer words or other methods sensitive on somewhat larger spatial scales [6–8]. As it turns out, there is an astonishing amount of phylogenetic information contained in the frequencies of short segments of DNA. Naturally much interest has focused on the potential use of this in deducing phylogenetic relationships [6,7,9–13].

However, evolutionary patterns in the frequencies of short words are also of interest because of what they reveal about function in the genome. Within genomes, word-frequency variation has been linked to functional variation [14–16]. The time scale of evolutionary changes in these frequencies provides further evidence of a relationship to genome function. For example, interspecies distances constructed from di- and trinucleotide frequencies are smaller between mammals and chicken than between mammals and fish [4]. This is suggestive because in the amount of time since these species diverged, neutral sequence will have undergone multiple substitutions. The relatively slow rate we observe suggests the involvement of purifying selection at some level, and a relationship to functional processes. Karlin and Burge suggested that word-frequency variation reflects interspecies differences in processes such as DNA modification, replication, and repair [17]. In this case, the sequence differences would be a byproduct of changes in a functional process. The action of purifying selection would not be on the word frequencies directly, but rather on the functional processes, for example, DNA repair enzymes. Other possible explanations for word-frequency changes include sequence differences which are more directly functional, for example, reflecting differences in genomic signals for chromatin formation [18].

An important challenge is to try to sort out the different processes that may be influencing word-frequency variation [19]. Here we develop a method for doing that. The basic idea is to calculate interspecies distances which isolate the effects at particular word sizes. In doing so, we aim to isolate different functional categories. It seems likely that processes affecting the frequencies of 2-bp words may be different from those affecting longer words. Gene regulation provides an example of a process that affects the composition of longer words. The presence of transcription-factor binding sites in noncoding sequence is known to affect the composition of 8-bp words [14]. It would be of interest to know if word-frequency distances for 8-bp words scale with evolutionary time. The genomes of multicellular animals contain many thousands of regulatory elements [20,21]. Such elements are unevenly distributed, being more highly concentrated near genes, and especially in the promoter region upstream to genes. Here we make use of this fact by examining three different categories of noncoding genomic DNA: the promoter region 2 kb immediately upstream to genes, intronic sequence, and randomly selected intergenic sequence. We find that word-frequency distances do scale differently for different sequence categories and word sizes. In particular, word-frequency distances for 8-bp words scale with evolutionary time in promoter sequence, and more weakly in intronic sequence, but not in intergenic sequence.

## Results/Discussion

**Figure 1:**
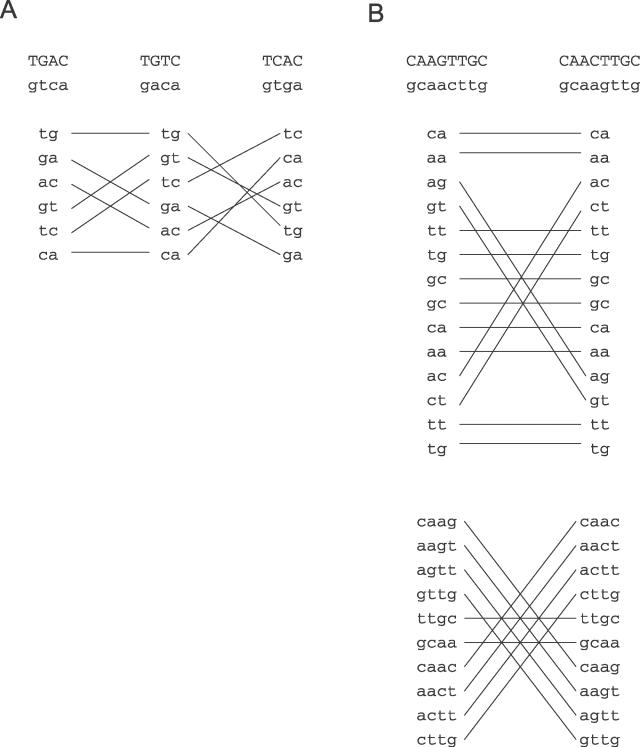
Examples of ISW Groups for 4-bp and 8-bp Words (A) An example of an iso GC/dinucleotide group in 4-bp words. This group consists of the tetranucleotides TGAC, TGTC, and TCAC. In addition to having the same GC content, these words (considering also their reverse complements which are written below them) share the same six dinucleotides. The dinucleotide composition of each word is written below it with lines showing that the same dinucleotides are present in all three words. (B) An example of an iso GC/di/tetranucleotide group in 8-bp words. The two words CAAGTTGC and CAACTTGC have the same GC content as well as sharing the same 14 dinucleotides and the same 10 tetranucleotides.

We sought to develop a method for determining interspecies distances in word frequencies which would remove the effect of smaller constituent words. For example, when we compare two species in terms of their composition of 4-bp words, we want to do so while removing any effects due to variation in the frequencies of 1-bp words (i.e., GC content) and 2-bp words. Our method relies on the fact that 4-bp words can be divided into groups where each group consists of words that have the same GC content and dinucleotide frequencies. We call these iso GC/dinucleotide groups of 4-bp words. One example of such a group is the set TGAC, TGTC, and TCAC. In addition to having the same GC content, these words (considering also their reverse complements) share the same six dinucleotides. This is illustrated in Figure 1A. There are 30 such groups of 4-bp words. We can similarly construct iso-GC groups for 2-bp words, and iso GC/di/tetranucleotide groups for 8-bp words. Figure 1B illustrates an 8-bp set, the words CAAGTTGC and CAACTTGC. These share GC, di, and tetranucleotide frequencies. There are 522 such groups for 8-bp words.

Next we calculate a distance based on a comparison of word frequencies within these iso-groups. To illustrate, let us continue with the 4-bp iso GC/dinucleotide group given in Figure 1A. For a given species, we count the number of occurrences of each of the three words including reverse complements. We then divide this by the sum of the three to produce three proportions. We iterate through all iso GC/dinucleotide groups this way, producing a vector of proportions. We then compare these vectors between species by calculating the Manhattan distance between them. We call this an iso-frequency word (IFW) distance. In this case it will reflect variation in the frequencies of 4-bp words, removing variation due to GC content and dinucleotide frequencies. The same principle can be applied to 2-bp and 8-bp words.

We applied our method to repeat-masked genomic sequence from 13 metazoan species. These species were chosen to provide a range of phylogenetic distances. They were: Homo sapiens (human), Mus musculus (mouse), Canis familiaris (dog), Monodelphis domestica (opossum), Gallus gallus (chicken), Xenopus tropicalis (frog), Danio rerio (zebrafish), Tetraodon nigroviridis (pufferfish), Ciona intestinalis (sea squirt), Ciona savignyi (sea squirt), Drosophila melanogaster (fly), Anopheles gambiae (mosquito), Caenorhabditis elegans (worm). For each species we obtained equal amounts of genomic DNA from each of four categories: 1) promoter sequence 2 kb upstream to each gene, 2) intronic sequence, 3) randomly chosen intergenic sequence, and 4) coding sequence. For each category within a species we obtained just over 5.5 megabases of nonrepetitive sequence. We then exhaustively counted all 2-bp, 4-bp, and 8-bp words in this sequence. We used these counts to calculate IFW distances between all possible pairs of species for each of the four categories of sequence. The resulting distances are given in Table S1.

**Figure 2:**
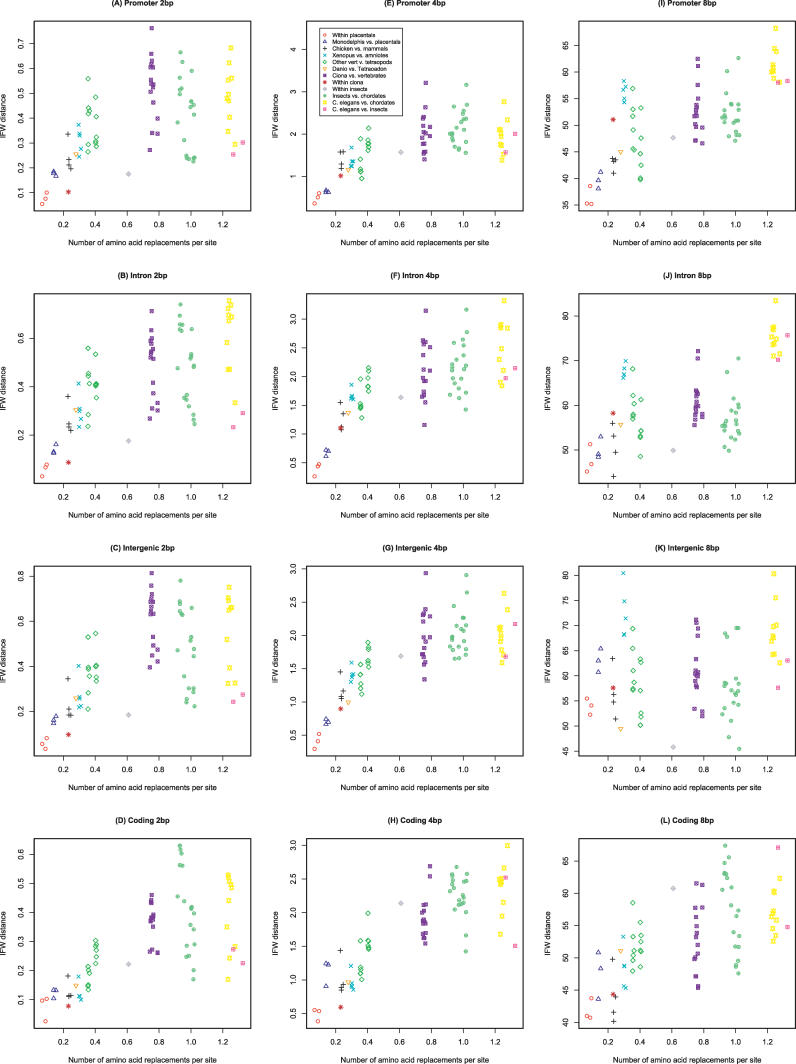
The Number of Amino Acid Replacements per Site versus IFW Distance Rows give results for promoter, intronic, intergenic, and coding sequence, respectively. Columns represent different word sizes. In each plot, a datapoint represents a species pair, and each plot contains all possible pairs for our 13 species. Note that the *y*-axis ranges vary from plot to plot.

Our interest is in seeing how IFW distances vary over evolutionary time. To examine this, it is useful to compare with a variable such as the rate of protein evolution, which is strongly associated with the degree of relatedness between species. We therefore calculated the number of amino acid replacements per site for all possible pairs of species in our set. We used 89 sets of unique best-reciprocal-hit orthologs, each set consisting of one protein from each of our 13 species. For these we downloaded high-scoring segment pair (HSP) protein alignments from Ensembl and used them to calculate the number of amino acid replacements per site for all possible pairs of our 13 species. Figure 2 contains plots of our IFW distances versus amino acid replacements for 2-bp, 4-bp, and 8-bp words in the four categories of sequence. A correlation between IFW distance and amino acid replacements suggests that word-frequency distances scale with evolutionary time.

Consistent with previous observations, Figure 2A–2H shows that IFW distances for 2-bp and 4-bp words do scale with evolutionary time. Species that are more closely related to each other have more similar word compositions. This is true for all four of our categories of sequence. The plots also suggest that this trend is strongest over short to medium phylogenetic distances. As one moves to the right in the plots, the IFW distances plateau. Another way to say this is that at large phylogenetic distances similarity in word frequencies has been reduced to a baseline level. In our data the scaling trend is strongest within chordates; however, this most likely does not represent anything distinctive about chordates. Rather it simply reflects the fact that the closest phylogenetic relationships in our data set all lie within chordates.

The most interesting result in Figure 2 is the difference between 2-bp and 4-bp words on one hand, and 8-bp words on the other. In the 8-bp case, word-frequency variation is different in different categories of sequence. In promoter sequence, Figure 2I reveals a positive association between IFW distances for 8-bp words and amino acid replacements. A similar though weaker trend can be seen in intronic sequence (Figure 2J). But in intergenic sequence, this association appears absent (Figure 2K).

We can test associations between distances statistically using a permutation test such as the Mantel test. For 8-bp words, the association between IFW distances and amino acid replacements is significant in promoter sequence (Mantel test, *p*-value = 0.002). For 8-bp words in intronic sequence, the association is also present (*p* = 0.023). But in intergenic sequence, it is not significant using our methods (*p* = 0.262). This is in contrast to 2-bp and 4-bp words that have significant trends in all noncoding categories (*p*-values < 0.001). Coding sequence also shows a significant association between IFW distance and amino acid replacements for 8-bp pair words (*p* = 0.005).

These results suggest that the patterns at different word sizes reflect different evolutionary processes. But before discussing this further we should consider one possible confounding factor. It is possible that our promoter and intron sequences contain some unannotated exons. As can be seen in Figure 2, coding sequence shows an association between 8-bp IFW distance and amino acid replacements per site. It is possible that the pattern we observe in promoters and introns is actually due to a higher density of surreptitious coding sequence in these regions compared with intergenic sequence. One argument against this is that the magnitude of the association between IFW distance and the number of amino acid replacements is comparable for coding and promoter sequence. If the effect in promoters were due to small numbers of unannotated exons, we would expect the association in promoters to be smaller. To further examine this issue, we repeated our procedure on a set of promoter sequences where we had eliminated all nucleotides which align with any mRNAs or ESTs. We limited this analysis to four tetrapod species with suitable annotations available: human, mouse, chicken, and frog. We found that the association between IFW distance and amino acid replacements remained (Figure S1).

An interesting question is what kind of evolutionary processes could produce different trends at different word sizes. In addressing this, we can begin by thinking about what we would expect from sequence evolving solely under the influence of random mutational drift. In such sequence, the rate of nucleotide substitution is fast enough that word composition similarities between species would decline to a baseline level relatively rapidly. In plots such as those of Figure 2, this would manifest itself as a lack of correlation between IFW distance and rate of protein evolution, because even closely related species would have large IFW distances between them. The majority of panels in Figure 2 in fact show something different, a slow steady scaling between IFW distances and evolutionary time. This suggests the involvement of purifying selection in some form.

Let us consider the patterns in 2-bp and 4-bp words in Figure 2A–2H. Karlin and Burge suggested that such patterns are due to evolutionary changes in processes such as DNA replication and repair [17]. A process such as DNA replication can produce certain biases in word composition as a byproduct of its operation. The idea is that in different species the process will produce different biases, with more closely related species having biases that are more similar. One attractive feature of this as an explanation is that replication and repair processes occur relatively evenly throughout the genome. This is consistent with the fact that we observe a correlation between IFW distance and amino acid replacements for all our categories of sequence at 2 bp and 4 bp. As noted above, the slow changes in word composition suggest purifying selection. However, in this scenario the purifying selection is indirect. It would not be acting on 2-bp and 4-bp word composition directly, but rather on the DNA replication or repair processes. Its action would tend to reduce the rate at which those processes diverge between species. As a result, the biases in short words caused by DNA replication and repair would also tend to change slowly over evolutionary time.

The case of 8-bp words in noncoding sequence likely reflects something different. What suggests this is the fact that for 8-bp words intergenic sequence behaves differently than our other noncoding categories. In intergenic sequence there is not a significant correlation between 8-bp IFW distance and the rate of protein evolution. A comparison of intergenic sequence in Figure 2K with promoter and intronic sequence in Figure 2I and 2J suggests a reason for this. In intergenic sequence, closely related species have relatively large 8-bp IFW distances. This suggests that the IFW composition of 8-bp words in intergenic sequence is less influenced by purifying selection than it is in promoters and introns.

What sort of process could account for this? Promoters and introns are both expected to have a higher density of regulatory elements than intergenic sequence. Purifying selection related to these elements could be slowing down the rate of change in word composition near genes. Because intergenic DNA has a lower density of such elements, the composition of 8-bp words would be free to change rapidly here. In this scenario the action of purifying selection is direct. Particular motifs are functional, and purifying selection slows the rate at which they change.

Just as amino acid replacements accumulate in proteins during the course of evolution, so changes in gene regulatory machinery are also likely to slowly accumulate. One level at which this might occur is in the frequency of particular transcription-factor binding motifs. Particular motifs may become more or less common over evolutionary time. Considered in aggregate this will result in a slow, steady increase in word-frequency distances. A related possibility is that changes in binding proteins themselves might necessitate genomewide changes in the sites they bind to. And of course the genome has many other methods of regulating genes. Changes in processes related to chromatin or the transcriptional unzipping of DNA could also be involved. Such processes depend on the interactions between proteins and particular patterns in DNA, and evolutionary changes in these interactions would affect word composition. Thus, an interesting possibility is that the slow changes in the composition of 8-bp words in our data reflect changes in the language of gene regulation.

## Materials and Methods

For each of 13 metazoan species, we obtained genomic sequence from version 39 of Ensembl (http://www.ensembl.org). The species used were listed above. We used the Ensembl PERL API to download four different categories of genomic sequence. In all sequences, repeats were masked by Repeat Masker [22]. First, we took sequence upstream to the transcription start of each gene. We took either 2 kb or as much as could be taken until we encountered the next gene. We next downloaded a set of intronic sequences. For each gene with multiple exons, we took up to 2 kb of intronic sequence. To do this we iterated through the introns in random order, taking randomly selected segments of sequence up to 2 kb in length. Once we had 2 kb from a particular gene, we moved on to the next. A third sequence set consisted of the Ensembl coding sequences for each species. A fourth set consisted of randomly selected intergenic sequence. For each species we downloaded the complete coordinates of every chromosome (or the top-level coordinate system of the assembly). We imagined lining up each of these end to end, and took a random sample from among all 2-kb segments, eliminating those which fell within 2 kb of an Ensembl gene. We then downloaded sequence corresponding to these from Ensembl. For the three noncoding sets, we masked sequences that were annotated as exons by Genescan or SNAP.

It was desirable to have a comparable sample size for different species and different categories of sequence. From the sequence we downloaded, we took random samples such that each category of sequence within each species had just over 5.5 megabases of nonrepetitive sequence. We then exhaustively counted all 2-bp, 4-bp, and 8-bp words in these sequences, and in their reverse complements, using a Python script.

To identify iso GC/di/tetranucleotide groups for 4-bp and 8-bp words, we used a script that we wrote in R [23]. There are 30 iso GC/dinucleotide groups of 4-bp words: 24 consisting of two words each, and six consisting of three words. There are 522 iso GC/di/tetranucleotide groups of 8-bp words: 516 consisting of two words each, and six consisting of three words. Note that our choice of word sizes reflects the nature of the method. There are, for example, no iso GC/di/tetranucleotide groups of 7-bp words. Also note that as we move to larger word sizes, the number of words belonging to these iso-groups becomes an increasingly smaller proportion of all possible words, which presents a technical barrier to the analysis of word sizes greater than eight.

We compare our IFW distances with amino acid replacement distances that we obtained as follows. First we used orthology information from version 39 of Ensembl to identify 89 ortholog sets. Each set consisted of proteins (one from each of our 13 species) that were annotated by Ensembl as one-to-one orthologs with each other. Then for each pairwise comparison in this set, we obtained the HSP protein alignment from Ensembl via the PERL API. We then calculated the amino acid replacement distances (corrected for multiple hits) between every two species using the 89 HSPs together. To do this we used PAML [24] with the substitution matrix of Jones et al. [25].

We tested the statistical significance of associations between IFW distances and amino acid replacements using the Mantel test with 100,000 permutations.

To make sure our result was not an artifact of surreptitious coding sequence, we repeated it with more stringent efforts to remove potential coding sequence. We did this in human, mouse, chicken, and frog, using the table browser Web site of the University of California at Santa Cruz (http://genome.ucsc.edu/). We first made a custom track consisting of 2 kb upstream to RefSeq genes in each species (in Xenopus we used gene annotations from JGI). We then did a bp-wise intersection of this with the complement of the following tracks: species-specific and xeno mRNA, the species-specific ESTs, ensembl genes, simple repeats, and repeat masker. With this sequence we then repeated the analysis as described above.

## Supporting Information

Figure S1 Protein Divergence versus IFW Distance Removing Nucleotides which Align with mRNAs and ESTs This is for human, mouse, chicken, and frog in promoter sequence using 8-bp words.
(4 KB PDF)

Table S1 IFW Distances for Promoter, Intronic, Coding, and Intergenic Sequences for 2-bp, 4-bp, and 8-bp Words (123 KB PDF)

## Abbreviations

HSP: high-scoring segment pair
IFW: iso-frequency word (distance)
