# Supplementary material for: The Evolution of Word Composition in Metazoan Promoter Sequence
Source: PLoS Comput Biol. 2006 Nov 3;2(11):e150. doi: 10.1371/journal.pcbi.0020150 (PMC1630712; doi:10.1371/journal.pcbi.0020150)
Supplement: Supplementary file 1 [file pcbi.0020150.sg001.pdf]

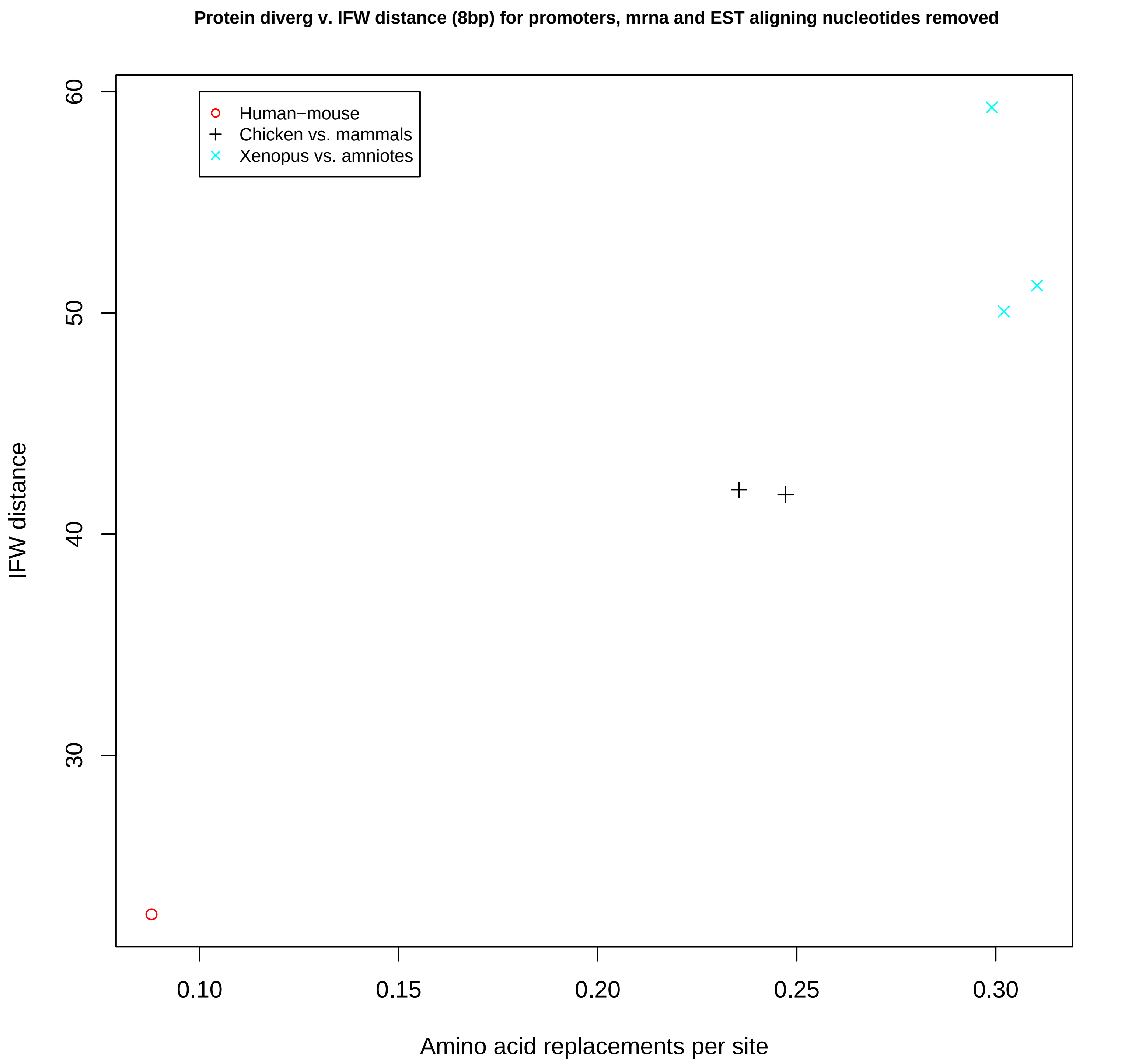
Protein diverg v. IFW distance (8bp) for promoters, mrna and EST aligning nucleotides removed
Human−mouse
Chicken vs. mammals
Xenopus vs. amniotes
IFW distance
30
40
50
60
0.10
0.15
0.20
0.25
0.30
Amino acid replacements per site
