# Supplementary material for: The Evolution of Word Composition in Metazoan Promoter Sequence
Source: PLoS Comput Biol. 2006 Nov 3;2(11):e150. doi: 10.1371/journal.pcbi.0020150 (PMC1630712; doi:10.1371/journal.pcbi.0020150)
Supplement: Supplementary file 2 [file pcbi.0020150.st001.pdf]

Supplementary Table S1 — IFW distances

Upstream_2_bp

	hs	mm	cf	md	gg	xt	dr	tn	ci	cs	dm	ag
mm	0.0755											
cf	0.0546	0.1005										
md	0.1774	0.1669	0.1846									
gg	0.2112	0.1965	0.2338	0.3356								
xt	0.3298	0.2766	0.3380	0.3732	0.2450							
dr	0.4411	0.4292	0.4178	0.5588	0.2941	0.2642						
tn	0.3072	0.3235	0.3000	0.4846	0.2864	0.4059	0.2550					
ci	0.6311	0.6167	0.6052	0.7622	0.6043	0.5242	0.3403	0.3988				
cs	0.5384	0.5546	0.5066	0.6592	0.5357	0.4631	0.2717	0.3373	0.1030			
dm	0.4681	0.4667	0.4449	0.5907	0.4142	0.4536	0.2355	0.2414	0.2389	0.2256		
ag	0.5143	0.5629	0.4965	0.6651	0.5203	0.6264	0.3828	0.3112	0.2389	0.2494	0.1750	
ce	0.4963	0.5535	0.4700	0.6228	0.5613	0.6831	0.4787	0.2948	0.4037	0.3467	0.3021	0.2538

Intergenic_2_bp

	hs	mm	cf	md	gg	xt	dr	tn	ci	cs	dm	ag
mm	0.0337											
cf	0.0554	0.0810										
md	0.1609	0.1777	0.1469									
gg	0.1838	0.1833	0.2106	0.3447								
xt	0.2565	0.2242	0.2644	0.4018	0.2180							
dr	0.3874	0.3562	0.3954	0.5298	0.2821	0.2101						
tn	0.4001	0.4041	0.3989	0.5458	0.3346	0.3516	0.2582					
ci	0.7199	0.7005	0.6862	0.8135	0.6860	0.5305	0.4483	0.4740				
cs	0.6646	0.6452	0.6309	0.7582	0.6333	0.4926	0.3956	0.4213	0.0969			
dm	0.5296	0.5301	0.5137	0.6590	0.4767	0.4448	0.3024	0.2227	0.3024	0.2856		
ag	0.6768	0.6885	0.6445	0.7803	0.6326	0.6284	0.4711	0.3558	0.2546	0.2401	0.1843	
ce	0.6925	0.7037	0.6501	0.7512	0.6604	0.6604	0.5191	0.3259	0.3931	0.3239	0.2747	0.2427

Coding_2_bp

	hs	mm	cf	md	gg	xt	dr	tn	ci	cs	dm	ag
mm	0.0254											
cf	0.0967	0.1029										
md	0.1331	0.1315	0.1039									
gg	0.1141	0.1139	0.1107	0.1814								
xt	0.1110	0.0996	0.1122	0.1795	0.1123							
dr	0.2016	0.1912	0.1342	0.2135	0.1507	0.1464						
tn	0.2831	0.2903	0.2245	0.3034	0.2475	0.2702	0.1486					
ci	0.3802	0.3827	0.3695	0.4600	0.3755	0.3515	0.2723	0.2620				
cs	0.4327	0.4361	0.3715	0.4427	0.3925	0.3875	0.2654	0.2606	0.0778			
dm	0.4129	0.4164	0.3573	0.4188	0.3971	0.3419	0.2508	0.2904	0.2014	0.1701		
ag	0.6302	0.6298	0.5631	0.6160	0.6032	0.5611	0.4554	0.4389	0.2854	0.2473	0.2221	
ce	0.5217	0.5288	0.4413	0.5069	0.4855	0.4967	0.3503	0.2826	0.2432	0.1699	0.2254	0.2732

IFW Distances

Intron_2_bp

	hs	mm	cf	md	gg	xt	dr	tn	ci	cs	dm	ag
mm	0.0672											
cf	0.0297	0.0786										
md	0.1260	0.1615	0.1309									
gg	0.2339	0.2187	0.2467	0.3599								
xt	0.3088	0.2671	0.2985	0.4130	0.2342							
dr	0.4545	0.4128	0.4442	0.5587	0.2846	0.2360						
tn	0.4080	0.4098	0.4034	0.5340	0.3542	0.4124	0.3046					
ci	0.5994	0.5773	0.5892	0.7121	0.5555	0.4164	0.3101	0.3333				
cs	0.5456	0.5384	0.5206	0.6330	0.5152	0.3728	0.2683	0.3020	0.0876			
dm	0.5246	0.5343	0.5162	0.6377	0.4870	0.4817	0.3190	0.2459	0.2636	0.2843		
ag	0.6573	0.6934	0.6368	0.7395	0.6303	0.6556	0.4762	0.3541	0.3660	0.3460	0.1763	
ce	0.6958	0.7232	0.6719	0.7550	0.6879	0.7370	0.5822	0.3337	0.4720	0.4712	0.2908	0.2327

Upstream_4_bp

	hs	mm	cf	md	gg	xt	dr	tn	ci	cs	dm	ag
mm	0.5050											
cf	0.3620	0.6020										
md	0.6220	0.6280	0.6670									
gg	1.2920	1.5800	1.1900	1.5750								
xt	1.3540	1.3570	1.2390	1.2480	1.6800							
dr	1.1190	0.9510	1.1070	1.1720	1.8890	1.4060						
tn	1.7660	1.6950	1.6150	1.8580	2.1400	1.7780	1.1590					
ci	1.7730	1.5380	1.7670	1.5770	2.6330	1.5830	1.4040	1.9520				
cs	2.4060	2.1950	2.3690	2.0490	3.2100	2.0180	1.9120	2.1690	1.0150			
dm	2.5500	2.3470	2.4790	2.1010	3.1630	2.3630	2.2840	2.6920	1.8140	1.5570		
ag	2.0190	1.9910	1.9080	1.8490	2.6500	1.7010	2.0070	2.1490	1.6610	1.6310	1.5720	
ce	2.0150	2.1080	1.9450	1.7330	2.7640	1.5230	2.1010	2.3360	1.3860	1.7590	2.0060	1.5670

Intergenic_4_bp

	hs	mm	cf	md	gg	xt	dr	tn	ci	cs	dm	ag
mm	0.4090											
cf	0.2950	0.5190										
md	0.6650	0.6980	0.7410									
gg	1.0500	1.1640	1.0820	1.4520								
xt	1.4060	1.4190	1.3710	1.3020	1.5910							
dr	1.2710	1.1170	1.2030	1.4130	1.5630	1.4090						
tn	1.6200	1.5250	1.5840	1.8950	1.8230	1.7910	0.9960					
ci	1.8110	1.6750	1.7180	1.5630	2.3930	1.5970	1.3390	1.9720				
cs	2.3280	2.2090	2.3070	1.9710	2.9360	1.9070	1.7170	2.2860	0.8970			
dm	2.2650	2.0700	2.2660	1.9140	2.9070	2.1520	2.0960	2.6460	1.7930	1.7100		
ag	1.9800	1.9710	1.9350	1.7810	2.4430	1.6500	1.8340	2.1450	1.6570	1.8310	1.6890	
ce	2.0340	2.1240	1.9560	1.7820	2.6320	1.7080	2.1060	2.3860	1.5900	1.9090	2.1710	1.6820

IFW Distances

Coding_4_bp

	hs	mm	cf	md	gg	xt	dr	tn	ci	cs	dm	ag
mm	0.3850											
cf	0.5510	0.5360										
md	1.2430	1.2250	0.9040									
gg	0.8480	0.9310	0.8840	1.4360								
xt	0.8970	0.8540	0.9520	1.2080	0.9140							
dr	1.1520	1.0060	1.0980	1.5810	1.1880	1.0940						
tn	1.5860	1.4570	1.5090	1.9900	1.4780	1.5780	0.9670					
ci	1.6210	1.6200	1.6830	2.0280	1.8380	1.5410	1.8620	2.5380				
cs	1.8960	1.8350	1.8770	2.1130	2.1220	1.6970	2.0000	2.6890	0.5950			
dm	2.2220	2.1450	2.2590	2.4590	2.4710	2.0070	2.1250	2.5760	1.6610	1.4230		
ag	2.5450	2.5690	2.4220	2.4810	2.3650	2.0460	2.3180	2.6780	2.1830	2.1150	2.1380	
ce	2.4460	2.4200	2.5060	2.4570	2.6640	2.1520	2.4930	2.9950	1.9470	1.6780	1.5050	2.5200

Intron_4_bp

	hs	mm	cf	md	gg	xt	dr	tn	ci	cs	dm	ag
mm	0.4380											
cf	0.2630	0.4740										
md	0.6110	0.7010	0.7210									
gg	1.1230	1.3510	1.0750	1.5490								
xt	1.6650	1.6030	1.6400	1.6170	1.8570							
dr	1.4870	1.2820	1.4650	1.4470	1.9570	1.5280						
tn	1.8310	1.7460	1.8230	1.9740	2.1530	2.0960	1.3700					
ci	1.9210	1.7360	1.9780	1.6760	2.6020	1.5520	1.1590	2.1030				
cs	2.5610	2.3740	2.6290	2.1220	3.1460	1.9250	1.6510	2.5110	1.1050			
dm	2.5430	2.3690	2.6440	2.1280	3.1650	2.1910	1.9640	2.7740	1.7200	1.4250		
ag	2.1590	2.1080	2.2130	1.9700	2.5880	1.8830	1.6840	2.2950	1.6250	1.7940	1.6370	
ce	2.8480	2.8810	2.9050	2.4850	3.3210	2.1090	2.2980	2.8440	1.8400	1.9000	2.1460	1.9740

Upstream_8_bp

	hs	mm	cf	md	gg	xt	dr	tn	ci	cs	dm	ag
mm	38.6000											
cf	35.3000	35.2000										
md	39.6000	41.2000	38.1000									
gg	43.2000	43.5000	41.0000	43.7000								
xt	55.0000	57.3000	54.3000	56.7000	58.3000							
dr	49.1000	45.4000	45.6000	51.7000	53.0000	56.9000						
tn	40.1000	42.5000	39.8000	44.7000	47.6000	53.3000	45.0000					
ci	51.8000	53.0000	50.2000	53.8000	57.6000	62.5000	53.4000	49.6000				
cs	49.7000	47.2000	47.1000	52.3000	55.0000	61.1000	51.7000	46.6000	51.1000			
dm	48.8000	50.8000	47.1000	48.2000	52.9000	62.6000	50.5000	48.1000	53.9000	50.9000		
ag	51.9000	52.8000	50.6000	51.4000	56.0000	60.2000	51.5000	47.9000	53.9000	54.0000	47.7000	
ce	60.9000	61.4000	60.2000	58.8000	63.9000	68.2000	60.0000	58.1000	64.4000	60.2000	58.3000	58.0000

IFW Distances

Intergenic_8_bp

	hs	mm	cf	md	gg	xt	dr	tn	ci	cs	dm	ag
mm	52.3000											
cf	55.5000	54.1000										
md	63.0000	65.4000	60.7000									
gg	56.3000	51.4000	54.8000	63.4000								
xt	74.8000	71.4000	68.2000	80.4000	68.3000							
dr	61.0000	58.7000	57.4000	69.4000	57.2000	65.5000						
tn	57.1000	52.6000	50.2000	63.3000	51.8000	62.7000	49.5000					
ci	60.1000	58.1000	60.4000	70.6000	59.9000	68.0000	57.7000	52.9000				
cs	63.3000	61.1000	59.0000	71.2000	60.7000	69.4000	53.4000	52.0000	57.6000			
dm	59.5000	56.2000	56.5000	69.5000	58.4000	69.5000	54.7000	45.5000	54.2000	56.9000		
ag	58.6000	58.0000	53.6000	68.4000	58.5000	67.8000	52.3000	47.8000	57.1000	51.1000	45.8000	
ce	69.8000	67.9000	67.6000	80.3000	70.1000	75.5000	66.9000	62.6000	64.3000	64.2000	63.0000	57.6000

Coding_8_bp

	hs	mm	cf	md	gg	xt	dr	tn	ci	cs	dm	ag
mm	40.7000											
cf	41.0000	43.7000										
md	50.8000	48.3000	43.6000									
gg	40.2000	44.0000	41.6000	49.8000								
xt	48.8000	45.3000	48.7000	53.3000	45.6000							
dr	50.4000	51.1000	53.3000	58.5000	49.6000	48.0000						
tn	51.2000	53.5000	48.6000	55.5000	51.0000	52.5000	51.1000					
ci	54.9000	53.2000	57.8000	61.5000	52.0000	50.7000	53.9000	61.3000				
cs	47.1000	47.1000	50.1000	56.3000	45.6000	45.4000	49.8000	57.8000	44.3000			
dm	48.9000	48.6000	51.8000	56.5000	51.7000	47.6000	54.0000	53.4000	57.3000	49.6000		
ag	63.2000	63.0000	64.7000	67.4000	63.0000	62.4000	60.5000	65.6000	60.9000	58.1000	60.8000	
ce	52.6000	56.9000	57.3000	60.3000	55.8000	53.4000	56.4000	62.3000	60.1000	54.5000	54.8000	67.1000

Intron_8_bp

	hs	mm	cf	md	gg	xt	dr	tn	ci	cs	dm	ag
mm	51.3000											
cf	45.2000	46.8000										
md	49.1000	53.0000	48.4000									
gg	53.1000	49.5000	44.1000	56.0000								
xt	68.3000	69.9000	66.9000	66.1000	66.9000							
dr	58.0000	60.4000	57.6000	57.0000	62.2000	68.2000						
tn	52.8000	54.2000	48.5000	53.1000	54.3000	61.3000	55.7000					
ci	60.8000	63.3000	56.8000	62.0000	62.6000	70.5000	63.0000	57.4000				
cs	59.8000	59.4000	57.9000	58.6000	59.8000	72.1000	55.6000	58.1000	58.2000			
dm	56.6000	58.4000	52.3000	55.8000	59.5000	70.5000	61.5000	53.7000	60.2000	54.2000		
ag	55.8000	54.4000	50.7000	55.3000	56.5000	67.4000	55.4000	49.8000	58.8000	52.8000	49.9000	
ce	76.9000	73.6000	71.0000	77.5000	74.9000	83.4000	75.3000	71.5000	73.9000	77.3000	75.7000	70.2000
